# Tertiary Lymphoid Organs at the Center Stage of Skin's Humoral Immunity

**DOI:** 10.1111/imr.70061

**Published:** 2025-09-05

**Authors:** Inta Gribonika

**Affiliations:** ^1^ Laboratory of Barrier Immunity, Division of Molecular Hematology, Department of Laboratory Medicine Faculty of Medicine, Lund University Lund Sweden

**Keywords:** antibodies, B cells, microbiota, skin, T cells, tertiary lymphoid organs

## Abstract

The skin is the outermost organ that serves as the host's live, microbiota‐inhabited physical border, evolved to cope with continuous confrontation by a wide variety of environmental elements. This dynamic borderline is prone to injury and damage. Therefore, to deliver on the critical demands for protection, skin is tightly associated with innate and adaptive defense mechanisms that ensure homeostatic tissue barrier integrity. We recently described the skin's ability to form its own autonomous and protective immune response independently of known professional organs. Cutaneous immunocompetence was achieved through the formation of dermal tertiary lymphoid organs (TLOs) that provide protective humoral activity similar to the classical germinal center reaction in the lymph node. This response was mediated by cutaneous microbiota uncoupled from inflammatory signals and positioned within the healthy skin. Our findings illustrate the power of non‐inflammatory host‐microbiota interaction and open a door for reevaluation of topical disease development and progression. A detailed understanding of highly coordinated tissue‐specific determinants that facilitate local antibody response may provide innovative solutions in skin health care and therapies. In this review, I elaborate on our findings and argue for TLO's importance in the host's immune arsenal, which is at the very center of skin's humoral immunity.

## 1 Introduction

The mucocutaneous barrier separates the host and the outside environment [1]. It is continuously exposed to diverse exogenous antigens, including those of pathogenic origin. Barrier tissues are also primary sites for injuries and inflammation; each of these settings offers an opportunity for opportunistic pathogens to cross the host's borders. In addition to environmental stimuli, trillions of commensals populate the tissue, demanding symbiotic relationships while simultaneously controlling hosts' physiology [1, 2, 3]. However, coexistence with microbes also represents a major liability for the host. Indeed, most organisms colonizing the gut, skin, or lungs may potentially cause infection. Whether equipped with virulence factors or not, defined microbiota members can acquire pathogenic properties in the context of immunosuppression, indwelling medical devices such as catheters, or in premature infants [4]. This makes the mucocutaneous barrier one of the most vulnerable borders requiring a special type of structural and immunological armament to ensure host defense, safety, and protection.

All mucosal barrier sites are thoroughly guarded by immunoglobulins that form the most effective first line of immune defense. For instance, in the gastrointestinal tract, the largest mucosal and immunological barrier organ, coexistence with the microbiota is controlled by a sophisticated network of structural and immune factors, including immunoglobulin production [1, 3, 5]. Secretory IgA plays a crucial role in maintaining optimal host‐microbiota symbiosis in the gut by keeping the microbiota at a safe distance from the epithelium, thereby preventing microbial translocation and ensuring efficient gut colonization with host‐beneficial bacterial communities [6]. This is especially crucial due to the high biomass and diverse food antigens that must be controlled. A normal, healthy adult produces more IgA than all other immunoglobulin isotypes combined [7]. It is estimated that IgA antibodies are secreted at a rate of 60 mg/kg of body weight per day [7, 8] in specialized gut‐associated lymphoid tissues (GALT) that include isolated lymphoid follicles and secondary lymphoid organs called Peyer's patches found at the lining of the intestine. However, how such a dangerous necessity of coexistence with microbes is controlled at other barrier sites, and more particularly at those with low biomass, such as the skin, remains unclear.

Unlike mucous tissue, the cutaneous barrier is safeguarded by a very special physical fortress called stratum corneum—the outermost layer of the epidermis formed of terminally differentiated keratinocytes [1, 2, 9]. This keratinized barrier is vital for keeping water from evaporating out of the body and preventing pathogens from entering; it makes the skin appear barren, dry, and hostile to the naked eye—nothing more than a passive shield [9]. However, as it is the most exposed barrier tissue to elements and, in addition to the above‐mentioned challenges, requires protection from daily physicochemical stresses, nature has provided the skin with a unique collection of immune cells to deal with these special demands [1, 10]. The cutaneous immune defense has been primarily described by innate immunity and adaptive cell‐mediated immunity, with CD4^+^ and CD8^+^ T cells taking center stage and developing in skin‐associated lymphoid tissues (SALT), sometimes called the skin immune system, such as skin‐draining lymph nodes and spleen [10]. Circulation directs these antigen‐specific cells to the cutaneous compartment, where T cells establish residence dispersed evenly through the tissue and respond to commensal colonization, cosmetic applications, wound healing, tissue pathology, and cutaneous vaccination [10]. Yet, whether skin could form more organized lymph node‐like structures, such as in the GALT, and respond with immunoglobulin production to control various environmental and microbial challenges is a matter of debate.

We recently demonstrated that non‐inflammatory murine skin colonization with common human skin commensal *Staphylococcus epidermidis* profoundly modulates the skin immune landscape by inducing TLOs that support germinal center (GC)‐like dermal structures and antibody production [11]. Similarly to the known formation of tertiary lymphoid structures (TLS) at sites of chronic inflammation [12, 13], microbiota‐induced TLOs contained B cells and T cells of GC phenotype and function, dendritic cells, stromal components, and high endothelial venules [11]. Although dermal TLO induction was paralleled by classical GC induction in skin‐draining lymph nodes, it was preserved in lymph node‐deficient Lt⍺^−/−^ mice, albeit lower in magnitude, highlighting that under critical conditions, the skin can take the initiative to support the humoral response as the first line of immune defense [11]. Indeed, antibodies generated in this model were as effective at controlling barrier breach and systemic infections as those from wild‐type controls, emphasizing that a highly coordinated skin‐intrinsic immune cell landscape in host‐microbiota cooperation can generate a much stronger immune response than previously anticipated [11, 14]. It opens a door to a novel field of cutaneous humoral immunity with exciting possibilities for a better understanding of host‐microbiota interaction, cutaneous barrier surveillance and protection, skin disease etiology, and potential development of innovative topical biotherapeutic applications for patients. In this review, I elaborate on critical factors that we have identified for dermal TLO formation, discuss reported evidence in support of dermal TLO formation within the human skin, and consider potential clinical applications based on dermal TLOs as a platform for developing new therapeutic strategies.

## 2 Microbiota Control of Dermal TLO Formation

The skin immune system has evolved in the context of its constitutive exposure to a complex microbiota. While the commensals seed all barrier tissues at birth and early in life, the host response is wired into a broad tolerization of adaptive immunity to early symbionts serving the important purpose of limiting tissue inflammation to a core microbiome acquired at birth [11, 15]. It is no surprise that microbiota has therefore been primarily viewed as a passive consortium of microbes that do not engage in the modulation of adaptive immune processes directed at commensal antigens or exogenous proinflammatory signals. Yet, barrier tissues remain permissible to new symbionts throughout the lifespan. Simple actions such as a change of diet, relocation, having a partner, starting a family, or the inevitable course of aging can all lead to changes in the composition of microbiota in adult life, with some commensals being lost, and others—gained [16]. Indeed, we found that skin autonomous production of commensal‐specific antibodies occurs in response to a new microbe acquired at the adult stage and not to microbes acquired early in life [11]. This indicates that tissue intrinsic adaptive immunity simultaneously serves as a border patrol and health insurance only after a cutaneous barrier is established with its core residents and infrastructure, meaning that the physiological niche and maturation status of tissue immune cells are prerequisites for effective humoral defense.

Skin adaptation to microbial symbiosis is associated with its autonomous ability to sustain local humoral responses through *de novo* dermal GC‐like TLO formation [11]. Using the lymph node‐deficient Lt⍺^−/−^ model, we could show that skin is self‐sufficient and can supply all components required to induce and sustain dermal TLOs. This was further confirmed in murine models with impaired cellular migration, such as in mice that lack C‐C chemokine receptor 7 (CCR7) – an important chemokine receptor for skin dendritic cell migration to the lymph node [17, 18]. The preserved ability of the skin to develop TLOs highlights the unique strategy employed by this tissue to coexist with its numerous environmental challenges both under a steady state and in the context of infection. While skin has long been thought to be devoid of B cells [19], recent studies have shown cutaneous B cell accumulation in health and disease [20]. Our work shows that B cells are skin residents that form the basis of TLOs in response to microbiota and undergo differentiation into plasma cells (PCs) via GC‐like education within the tissue [11].

**FIGURE 1:**
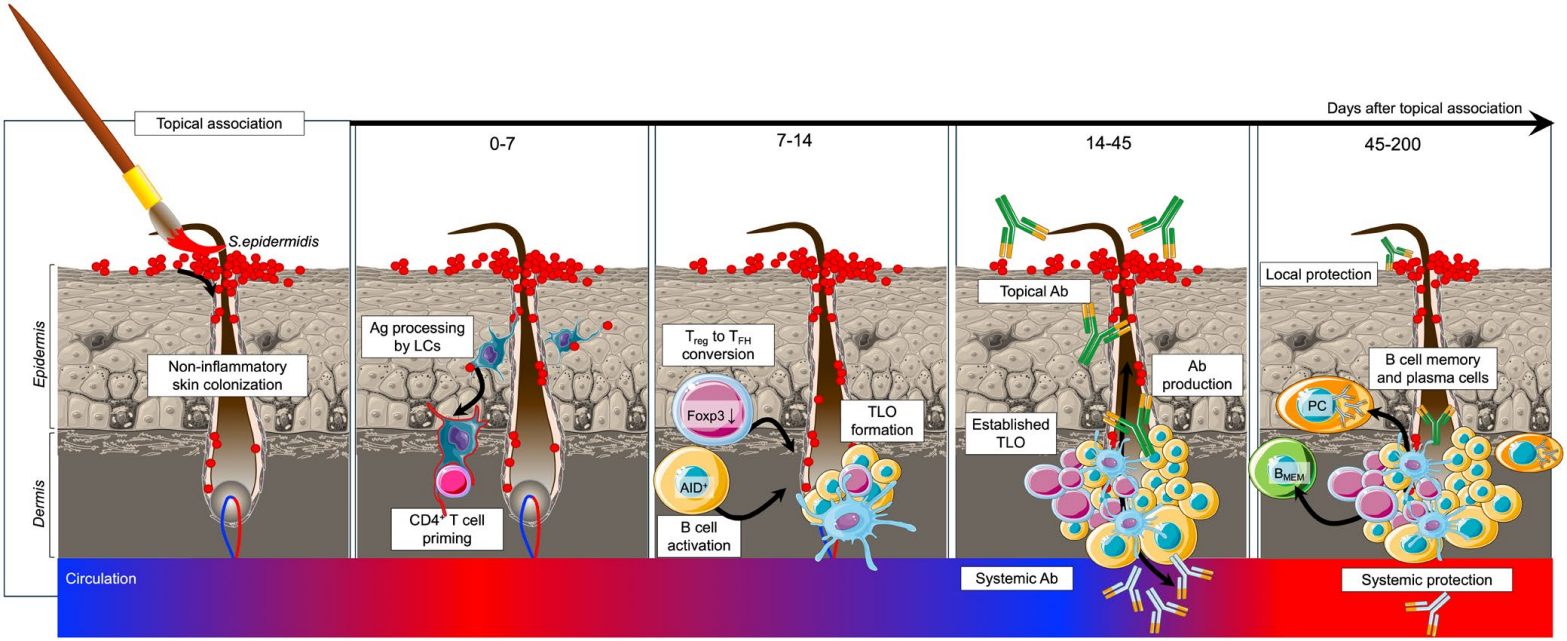
Dermal TLO formation in murine skin after non‐inflammatory association with a novel commensal. Topical association with *S. epidermidis* is a non‐invasive and non‐inflammatory process that can be described as a gentle painting of bacterial culture onto the skin. This homeostatic event establishes long‐term skin colonization with a new microbe especially concentrated within the hair follicle. In the first 7 days after association, Langerhans's cells (LCs) process and present the bacterial antigens to prime dermal CD4^+^ T cells, which leads to downmodulation of Foxp3 expression in T regulatory cells (T_reg_) and subsequent initiation of the T follicular helper cell (T_FH_) program. Dermal B cells are also activated, as evidenced by AID expression, and in the 2nd week post‐association, dermal TLOs start to emerge, fully forming by day 21. Germinal center (GC)‐like activity within TLOs generates commensal‐specific antibodies (Ab) that are secreted systemically in serum and topically onto the skin's surface. The antibody concentration in serum gradually increases from 14 to 45 days after which a stable serum titer is maintained for up to 200 days, possibly even longer. Dermal TLOs remain present during this timeframe, contributing to host protection by producing memory B cells (B_MEM_) and plasma cells (PCs) [11]. This figure was created in MS PowerPoint (v16.97.2) using graphical templates from Servier Medical Art under a Creative Commons license CC BY 4.0 (https://creativecommons.org/licenses/by/4.0/).

Microbiota‐induced dermal GC‐like structures are formed under non‐inflammatory settings, where, after a week of non‐invasive skin colonization with *S. epidermidis*, immune cells start to accumulate around the hair follicle, showing first signs of B cell activation and downmodulation of the suppressive T regulatory cell (T_reg_) program that gives rise to T follicular helper cells (T_FH_) (Figure 1). The dermal GC then evolves and fully matures 2 weeks later, and by day 45, substantial microbiota‐specific PC concentration is observed in the skin around the hair follicle. TLOs remain present in the skin for at least 6 months after colonization, ensuring stable antibody titers both locally in the tissue and systemically (Figure 1). The dominant immunoglobulin isotype produced within TLOs for *S. epidermidis* colonization is IgG, more specifically IgG2b and IgG2c [11]. In mice, these isotypes are more effective at activating the complement pathway and binding to cellular Fc‐receptors than IgG1 [21, 22], therefore providing superior protection with broad‐spectrum effector functions. In support of this argument, skin antibody responses alone and in the absence of secondary lymphoid structures were highly effective at controlling microbiota‐induced systemic infection [11], representing a powerful protective mechanism of host defense against any commensals that may turn into an invasive opportunistic microbe. In addition to their protective function, local antibody responses also controlled microbiota burden, thereby allowing for the establishment of long‐term symbiosis. Whether antibody effector function contributed to this phenomenon remains to be explored. We could also propose that, analogously to gut IgA [3, 6], skin antibodies may also control the localization and/or behavior of the skin microbiota. However, whether IgG2b and IgG2c can specifically access microbial niches and impact microbial function remains to be addressed.

It is important to emphasize the striking efficiency of the dermal TLOs to mount robust, specific, long‐lasting antibody responses under non‐inflammatory conditions and at a very low antigenic threshold. Our experiments showed that murine cohabitation with a colonized individual can trigger skin response in every adult in the closed shared space [11]. Such specialized cutaneous function may result from the unique microanatomical structures and antigen‐presenting cells associated with these responses. The skin microbiota primarily resides within the hair follicles, which are highly conserved structures endowed with numerous biological functions [23, 24, 25]. Hair follicles have also been shown to have more permissive cell adhesions than the rest of the epithelium, a feature that may allow microbiota‐derived antigens to be delivered to antigen‐presenting cells either via secretion and/or as outer membrane vesicles [26]. These appendages are associated with Langerhans cells (LC), which are specialized antigen‐presenting cells residing in the epidermis. Although LCs are known to self‐renew in the skin, hair follicle also serves as a reservoir for epidermal LC precursors [27, 28]. While LCs have been proposed to contribute to immunoregulation, as well as to adaptive immunity [29, 30, 31, 32, 33], there is little consensus regarding their dominant function. These cells form the densest network of antigen‐presenting cells in the body and are strategically positioned in the epidermis at the interface with the microbiota [34, 35]. We, however, demonstrated that in the context of non‐inflammatory skin colonization, one of the major functions of LCs is to regulate host‐microbiota interaction via their ability to promote local T_FH_ development and subsequent antibody responses [11].

Our work proposes that skin TLO's purpose to support T‐dependent antibody production may also derive, at least in part, from the ability of T_reg_ to convert into T_FH_ cells [11]. While LCs have been shown to engage with skin resident T_reg_ cells [31, 36] as well as induce T_FH_ program in skin‐draining lymph nodes [30], it is not clear whether LCs directly orchestrate downmodulation of the Foxp3 program in T_reg_ to give rise to T_FH_ cells. Nevertheless, CD4^+^ T cell instability in the tissue, particularly that of the gastrointestinal tract, has been reported, primarily focusing on the T_reg_—T_FH_—T_H_17 cell axis [37, 38, 39, 40]. For T_reg_ to undergo plasticity is a way to coordinate IgA response that maintains the commensalism with the microbiota in the gut [40, 41, 42, 43]. Indeed, mild yet persistent long‐term antigenic stimulation within the tissue appears to be crucial, as strong stimuli vaccination preferentially targets naïve cell population in lymphoid organs for T_FH_ induction [38]. Barrier tissues such as the skin are dominated by activated CD4^+^ T cells, and the naïve cell pool in the tissue is limited. Simultaneously, the skin harbors one of the highest frequencies and number of T_reg_ cells [15, 44, 45] and intriguingly, it has been shown that tissue T_reg_ cells have distinct transcriptome and clonally expanded TCR repertoires [45, 46, 47]. Indeed, a recent study elegantly demonstrated that across all major organs, tissue‐resident T_reg_ populations are united by a common phenotypic program indicative of extensive clonal sharing, that is shaped by age and microbiome and distinct from lymphoid counterparts [45]. One could speculate that the skin T_reg_ cells may be biased toward canonical microbial and cutaneous antigen specificities, allowing for the development of microbiota‐specific T_FH_ program. However, highly focused experiments are urgently needed to understand how the tissue‐resident T_reg_ repertoire could account for highly diverse microbiota‐derived antigens.

Together, our work shows that, similarly to rapid and effective humoral responses in secondary lymphoid organs and isolated lymphoid follicles at the mucosal barrier, the skin can also facilitate organized immune structures that provide effective tissue defense through TLO formation at a steady state. Whether dermal TLOs can be classified as permanent members of SALT remains to be addressed. Nevertheless, our observations indicate that once induced, TLO persistence is indefinite due to continuous commensal antigen availability [11].

## 3 Division of Labor Between Dermal TLOs and Skin‐Draining Lymph Node GCs

Skin is strategically positioned to face the outside environment while resting on a sophisticated grid of lymph nodes that are aimed to drain cutaneous antigens, including those of microbial origin, and to mount effective cellular responses [48, 49]. Our work shows that parallel to dermal TLO formation is also the induction of classical GCs in skin‐draining lymph nodes that support antibody production to colonizing commensal [11, 14]. Classically, T‐dependent humoral immunity forms via GC reaction in secondary lymphoid follicles within lymphoid organs. The unique feature of this process is that it allows for B cell education and subsequent differentiation, providing clonal expansion, affinity‐based selection, and somatic hypermutation in a dense, tightly controlled yet highly dynamic microenvironment [50]. Skin TLOs provide another venue for this type of response. At both sites, B cells expressed phenotypic GC markers such as T cell and B cell activation marker GL‐7, B‐cell lymphoma 6 (Bcl6), CD95, CD44, marker of proliferation Ki‐67, and activation‐induced cytidine deaminase (AID); however, BCR sequencing analysis did not confirm clonal relatedness between the dermal and lymph node compartments. Indeed, cutaneous B cells retained their GC phenotype even in the Lt⍺^−/−^ model that lacks skin‐draining lymph nodes, highlighting complementary yet diverged induction pathways that do not depend on each other [11].

In Lt⍺^−/−^ mice, dermal TLOs retained the wild‐type TLO characteristics, more specifically, the composition of GC T and B cells, class‐switched antibodies, and high endothelial venules encapsulating hair follicles that were associated with chemokine (C‐X‐C motif) ligand 13 (CXCL13) gradient and expression of complement receptors [11, 51, 52]. It is curious to think about what molecular tissue‐intrinsic pathways control TLO induction, especially because TLO efficiency at generating commensal microbiota‐specific humoral response was not compromised—it effectively eliminated systemic infection in the Lt⍺^−/−^ model [11]. The role of lymphotoxin signaling is known to be crucial for structural GC organization and B cell activation in lymphoid organs [53, 54, 55]. Even more, it has been identified as a critical factor for TLSs in chronic inflammatory settings [56, 57]. Despite scientific consensus and in support of our findings, published work by others demonstrates that lymphotoxin‐deficient mice are immunologically capable of providing some level of protective antigen‐specific responses, albeit with delayed kinetics. Prior studies using Lt⍺^−/−^ mice have shown that respiratory infection with a low dose of influenza virus [58] or murine gammaherpesvirus‐68 [59] can be cleared in these mice, reporting that neither LTα nor professional lymph nodes are absolutely required for the generation of effective immunity against viral infection. Although lymph nodes and GCs were absent, both studies showed that Lt⍺^−/−^ mice were able to induce virus‐specific CD8^+^ T cells and generate B cells that produced isotype‐switched virus‐specific antibodies [58, 59]. While it is unclear how adaptive immunity has been formed, these responses might have been induced in nasal‐associated lymphoid tissues, which are still present in lymph node‐deficient mice [60], or in the spleen. However, the probable source of immune induction in these mice is also the lung tissue itself, whereupon infection TLSs were formed, hosting B cells with GC phenotype [58]. Subsequent studies showed that these structures, in a lymphotoxin‐independent manner, mount effective anti‐viral immunity and retain immunologic memory [61, 62]. In line with these observations, another earlier study using a well‐established experimental (4‐hydroxy‐3‐nitrophenyl) acetyl‐ovalbumin (NP‐OVA) immunization model demonstrated that in Lt⍺^−/−^ mice systemic administration of high doses of NP‐OVA manifested somatic hypermutation of B cells leading to a high‐affinity anti‐NP IgG1 response similar to that observed in WT control mice [63]. Together, these reports demonstrate that in certain conditions, Lt⍺^−/−^ mice can mount effective antigen‐specific humoral immunity to infection, immunization, and at steady state independently of lymph nodes or classical GCs.

One factor that could potentially compensate for the lack of lymphotoxin activity and hence allow for TLO formation in this system is tumor necrosis factor (TNF); however, due to the cytokine relatedness, Lt⍺^−/−^ mice naturally seem to fail to produce physiological levels of TNF [64]. In addition, despite major immunological impairments, LTα and TNF double deficiency has been shown to allow for eventual adaptive immune response formation and clearance of vaccinia virus and lymphocytic choriomeningitis virus infections [65], demonstrating that other critical stimuli control adaptive immunity in lymph node‐deficient settings. Indeed, when we colonized TNF⍺^−/−^ mice, dermal TLO formation and commensal‐specific antibody response were preserved [11]. This suggests that the mechanism controlling TLO formation is fundamentally different from the known mechanism that controls GC formation in lymphoid tissues. It is essential to understand these inductive circuits to be able to control tissue response and assure host protection.

It is important to note that dermal TLOs differed from GCs not only in their inductive mechanism but also in humoral output—while the skin supported commensal‐specific IgG2b and IgG2c responses, lymph nodes supplied primarily IgG1 and IgG3 isotypes. We could show that the skin‐derived responses were fully capable of eliminating systemic infections—a feature that was shared with lymph node‐derived isotypes. In addition, skin‐derived antibodies effectively controlled skin bacterial burden, maintaining homeostatic equilibrium with pre‐existing skin colonists. It is not clear whether serum antibodies can access the cutaneous compartment in the murine host. Due to technical limitations, we were not able to address whether, in the absence of dermal TLOs, lymph node‐derived antibodies could be sufficient for local tissue protection and control of skin biomass. Nevertheless, this observation highlights segregation in antibody‐mediated functions [11].

Such dichotomy of responses among barrier and lymphoid compartments, coupled with the skin's autonomous ability to sustain antibody responses, may provide insights into the evolutionary perspective of humoral immunity. Skin‐associated B cells and antibody‐secreting cells have been discovered and characterized in the skin of lower vertebrates, such as teleost fish [66, 67]. Teleosts and mammalians diverged approximately 400 million years ago and do not have an immediate common ancestor [68, 69]. However, teleosts represent the most ancient bony vertebrates containing spleen, thymus, and SALT despite lacking lymph nodes and bone marrow [67, 70]. Skin composition in fish is significantly different, lacking keratinization, secreting mucous, and hosting diverse microbiota. It behaves as a mucosal surface; therefore, it is important to note that teleost SALT structurally resembles their GALT [66, 67]. Environmental pressure imposed by life in aquatic habitats forces tissue adaptation. In this context, the skin is in direct contact with water, adapting to the host demands of effective physical protection. Yet, cutaneous adaptation continues with evolution—after leaving the water medium, vertebrate skin lost its mucus production and underwent keratinization. Subsequently, mammalian skin developed hair follicles, sebaceous, and sweat glands. However, when terrestrial ancestors of aquatic and amphibious mammals such as cetaceans and hippopotamids returned to life in water approximately 50 million years ago [71], skin acquired further adaptations by developing an exceptionally thick epidermis with reduced hair follicles and skin glands [72, 73]—features controlling effective thermoregulation, mechanical protection, and survival [74]. Therefore, external factors imposed by the habitat are undeniable forces of evolution for barrier tissues. In teleost fish, humoral immune protection is segregated in IgM‐mediated systemic response and local tissue immunity ensured by IgT antibody—a specialized teleost mucosal immunoglobulin that predominantly coats skin microbiota and provides local tissue protection from skin infections [67, 75]. In the skin, IgT^+^ B cells were reported to be linked with mucosa‐associated lymphoid clusters, mimicking known features of mammalian GALT [67]. Even more, despite the fundamental differences in comparison to endothermic species, teleost fish were shown to mount effective systemic GC‐like responses upon infection [76].

These observations echo our findings in murine models [11]; therefore, it is probable that IgG2 antibodies in murine skin serve similar barrier functions to those of IgT antibodies in teleost fish or IgA antibodies in mammalian mucosa. While *S. epidermidis* skin colonization did not induce an IgA response, it remains to be investigated whether dermal TLOs can support IgA antibodies against other agents or in different mammalian species. Nevertheless, it appears that humoral immunity protects all barrier tissues under the guidance of primordially conserved principles. Thus, one could speculate that skin microbiota‐induced TLOs may represent a more primitive, ancient arm of immune system evolution maintained independently of lymph nodes as a means to coexist with symbionts.

## 4 Evidence of Cutaneous Humoral Immunity in Human

**FIGURE 2:**
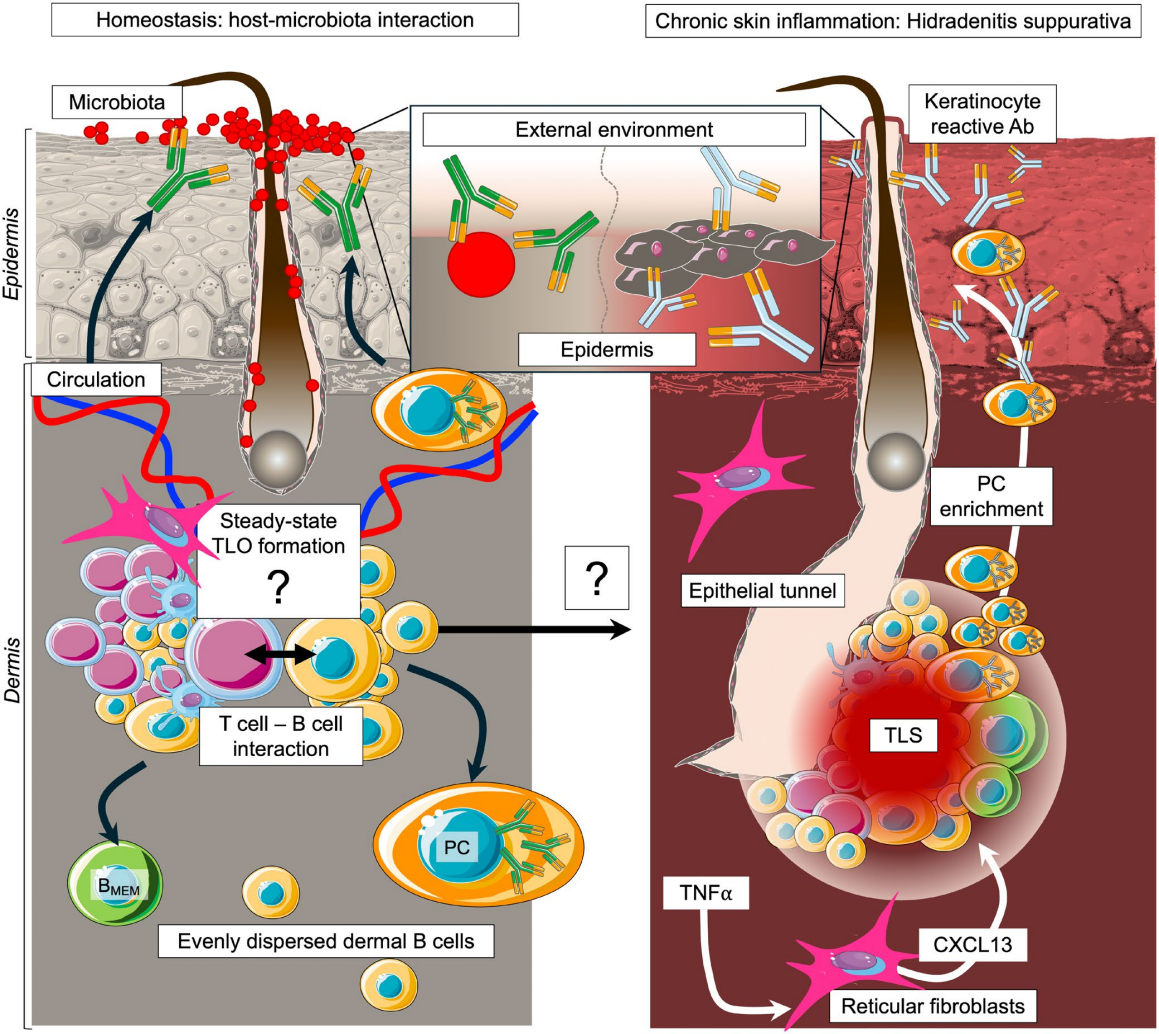
Humoral immunity in human skin. Microbiota in healthy human skin at homeostasis is coated with immunoglobulins [80]. It is believed that most of the topical antibodies come from the circulation; however, skin‐associated naïve B cells, class‐switched memory B cells (B_MEM_), and plasma cells (PCs) have been found evenly scattered throughout the dermis [78, 79]. It is not clear whether, at steady state, human skin supports T cell‐B cell interactions in TLOs, yet clinical evidence suggests that under chronic skin inflammation, such as in Hidradenitis suppurativa (HS), germinal center (GC)‐like TLSs are formed and facilitate local B cell expansion, somatic hypermutation, affinity maturation, and PC generation. HS is characterized by the formation of highly inflammatory intradermal epithelial tunnels (ETs) and pustular nodules. TLSs form adjacent to ETs in HS lesions to generate keratinocyte‐specific antibodies that extensively contribute to disease pathology. Recent work suggests that early TLS development is facilitated by TNF‐signaling to reticular fibroblasts that, in return, secrete CXCL13—a critical chemokine that acts as a chemoattractant, guiding the migration of B cells and T follicular helper cells to form GCs [12]. If homeostatic TLOs exist in human skin, then it is probable that a dysregulated response can lead to TLS development and chronic inflammation. More focused research must be performed to address this question. This figure was created in MS PowerPoint (v16.97.2) using graphical templates from Servier Medical Art under a Creative Commons license CC BY 4.0 (https://creativecommons.org/licenses/by/4.0/).

Initial histological conclusions of human skin immune landscape were drawn from analyzing healthy skin biopsies taken from the extremities and upper body—chest, shoulders, and abdomen—of patients with localized skin lesions [19]. B cells were not found, and hence, human skin was considered to lack the humoral arm of immunity [19]. Subsequent studies, however, demonstrated the existence of skin‐recirculating B cells [77] and, more recently, skin‐homing B cells [78, 79] that are evenly dispersed throughout the dermis at steady state (Figure 2). Yet, it is very problematic to make any firm conclusions about skin's immune composition from tiny biopsies with limited sample size simply because the skin is among the largest organs that lack uniform composition throughout the body [81]. For example, the skin of the face accommodates facial expressions and hence is very thin and flexible, while the skin of the heel must withstand continuous force and hence is thick and rigid [81]. It is widely accepted that the flat surface area of skin equals approximately 2 m^2^ [82]. However, if all appendages are considered, an estimate of total skin area increases to at least 30 m^2^ [83]. Across skin regions, the density and variety of skin glands and hair follicles vary considerably. This is important to consider because, although not directly exposed to the general external environment, the epithelial lining of hair follicles and skin glands is in contact with skin colonizing microbiota [23, 24, 25, 84].

Cutaneous microorganisms have evolved over millions of years to cope with external challenges that the skin is exposed to. Naturally, cellular and microbial composition differ greatly between the body sites of the skin [85, 86]. For example, the forearm hosts the most diverse set of microbes, while the least diverse community resides behind the ear [86]. Sebum‐rich zones such as the face, chest, and back predominantly host *Propionibacteria* and *Staphylococci* species, while in moist zones, *Corynebacteria* species dominate, followed by *Staphylococci* species [86]. In addition, microbiota abundance varies between regions in our body—using culture methods, it was estimated that the total microbial counts could be as low as 10^3^ in the driest areas such as legs to as much as 10^6^ per cm^2^ in the moistest skin areas such as hairy, warm axilla [87, 88]. Actual microbial abundance is probably much higher because traditional culture‐based methods allow only the detection of species that readily grow under standard laboratory conditions. Nevertheless, this estimate provides a trend where distinct skin regions could be as ecologically dissimilar as forests are to urban landscapes, influencing homeostatic immune responses and the need for B cells. Our work in murine models demonstrates the link between skin B cells and commensal microbiota [11]; hence, the cell enrichment could be better observed in skin areas with higher microbial diversity and overall abundance or a certain type of dominant microbial species. Therefore, judging cutaneous B cell content at homeostasis from a small biopsy is not representative of the entire organ. More thorough body‐wide cartography of skin immune cells is needed for a better estimation of the impact that humoral immunity has on the cutaneous barrier.

While B cells themselves have been scarcely observed in human skin, antibodies have been detected in ear wax [89], comedonal extracts [90], eccrine sweat [91, 92, 93, 94, 95, 96], and sebum [96]. In fact, all immunoglobulin isotypes, including secretory IgA, are secreted to the skin surface where they coat commensal microbiota at a steady state [80] (Figure 2). The origin of these antibodies is assumed to be systemic from serum, though in light of our findings, it is probable that a portion of human skin immunoglobulins is produced in the tissue via homeostatic TLOs [11, 90]. Topical antibody transfer could occur by mechanisms that range from mere diffusion and secretion into the pilosebaceous lumen to association with epidermal cells and subsequent epidermal shedding [90, 97, 98]. Yet, a detailed mechanism remains to be elucidated. A more concrete translocation pattern has been proposed for secretory IgA, which is particularly enriched in the axilla, where skin glands are very dense. The secretory component has been localized along the external surface membrane of mucous cells on the terminal segment of the eccrine sweat gland. It mediates endocytosis of dimeric IgA‐J chain complex across mucous cells and thus into the sweat [93, 95]. Eccrine sweat glands open directly onto the skin's surface and in humans can be found throughout the body, while in mice these glands are limited to the footpad [99]. As a result, structural differences in the skin between body sites and at the species level might dictate rules of antibody type and transport to the epidermal surface.

Chronic skin inflammation drives enhanced systemic antibody enrichment and B cell and PC accumulation in human skin [20, 100](Figure 2). For instance, acne patients produce large amounts of antigen‐specific antibodies found in serum and comedonal extracts against *Cutibacterium acnes*—a key agent in the development of acne vulgaris [90, 101, 102]. Atopic dermatitis patients have significantly elevated IgE titers in serum and eccrine sweat, contributing to skin pathology [91, 103]. IgA antibodies have been found in sweat gland tumors [94], but IgM memory B cells were identified in the skin infection site in early Lyme disease [104]. Interestingly, GC phenotype has been observed in human skin lymphomas—primary cutaneous follicle center lymphoma is characterized by BCL6^+^ B cells [105, 106], AID expression, and aberrant somatic hypermutation has been observed in a type of primary cutaneous large B‐cell lymphoma [107], but T_FH_ phenotype is characteristic of primary cutaneous follicular helper T‐cell lymphoma [108, 109]. In addition, inducible costimulator (ICOS)‐expressing T cells with a T_FH_‐like phenotype were shown to infiltrate the skin of patients with systemic sclerosis [110, 111, 112]. Systemic sclerosis is a chronic connective tissue disease characterized by the excessive deposition of extracellular matrix proteins in the skin and internal organs as a result of dysregulated tissue repair. While disease pathogenesis remains largely elusive, in an experimental murine model, ICOS^+^ T_FH_‐like cells preceded and accompanied skin scarring and fibrosis by promoting IL‐21‐dependent myofibroblast differentiation [110]. Indeed, anti‐ICOS or anti–IL‐21 treatment ameliorated disease in the mouse model by inhibiting inflammation and dermal fibrosis [110]. Therefore, targeting cutaneous T_FH_‐like cells in the skin might bring relief to scleroderma patients [110]. Together, these observations indicate that the human skin could support antibody responses and immune cells with an active GC‐like phenotype under an inflammatory setting. It is commonly accepted that skin inflammation drives immune cell infiltrates from circulation; however, reported observations of TLS in various inflammatory skin diseases question the existence of a local GC response that contributes to cutaneous pathology.

The pathological nature of cutaneous B cells associated with TLS was first reported in pemphigus vulgaris—a rare chronic skin disease where T‐dependent IgG autoantibodies inhibit cell‐to‐cell adhesions of keratinocytes and cause blister formation [113, 114, 115]. Local in situ B cell differentiation within TLS was represented by centroblast, plasmablast, and PC containment in these structures. TLS‐associated B cell populations also expressed differentiation‐defining transcription factors—B lymphocyte‐induced maturation protein‐1 (BLIMP‐1), interferon regulatory factor 4 (IRF4), and BCL‐6—and due to close contact with T cells could undergo local GC‐like reaction. Indeed, lesional B cells differed in BCR enrichment from their peripheral counterparts, confirming local clonal expansion in the skin [115].

More recently, TLS association with disease pathology was observed in hidradenitis suppurativa (HS)– a painful, long‐term skin condition that causes cutaneous abscesses and scarring in hair follicle‐ and sweat gland‐rich areas where skin rubs together, such as the axillae, inguinal and inner thigh regions, inframammary folds, and other similar sites [12, 13, 116] (Figure 2). In HS skin, B cells and PCs are dramatically increased compared to healthy controls, leading to an abnormally activated humoral response that causes dermal damage. Cutaneous B cells were shown to form follicle‐like structures within the dermis, suggestive of ectopic GCs [116], and more recent findings confirmed TLS association with HS lesions adjacent to disease‐characterizing epithelial tunnels [12, 13]. TLS backbone was formed of CXCL13‐expressing reticular fibroblasts that, in a TNF‐α‐dependent manner, orchestrated lymphocyte aggregation [12]. Within TLSs, organized interactions among activated and proliferative T_reg_, T_FH_ cells, B cells, and antigen‐presenting cells, and skin stroma took place to facilitate class switch recombination, somatic hypermutation, affinity maturation, and extensive clonal expansion of B cells that gave rise to PCs producing antibodies reactive to keratinocytes [12, 13] (Figure 2). The exact cause of HS is unclear, yet disease onset is attributed to the blockage, swelling, and eventual rupture of the hair follicle [117]. Of note, HS typically begins after puberty and disproportionately affects women, causing a significant negative impact on quality of life. Drawing parallels with homeostatic TLO formation around the hair follicle in response to microbiota in mice [11], it is intriguing to note that one of the factors attributed to multifaceted HS etiology is microbial colonization – skin microbiome imbalance and arrival of opportunistic colonists such as *Staphylococcus aureus* and members of intestinal microflora [117, 118]. The precise role of microbiota in disease onset is unknown; we can only speculate whether bacterial colonization of skin lesions is a primary cause, triggering factor, or secondary phenomenon of HS pathogenesis. Hypothetically, it is probable that, along with external factors and overactive immunity, dysregulated homeostatic dermal TLOs develop into chronic inflammatory TLSs, causing skin pathology (Figure 2). However, as described above, a detailed analysis of normal human skin must be conducted to verify if homeostatic TLOs are present in body areas that are most affected by HS lesions.

The presence of microbiota and TLSs has also been observed in melanoma tumors [78, 119, 120, 121, 122]. While microbiota‐specific T_FH_ cells were shown to induce TLS and provide anti‐tumor immunity against colorectal cancer in mice [123], the link between skin TLS formation and microbiota in skin malignancies has not been explored. Unlike HS or pemphigus vulgaris, an increased number of B cells and TLSs in melanoma are associated with enhanced anti‐tumor immunity and better patient prognosis [78, 124, 125]. For instance, TLSs rich in B cells and activated T cells were shown to directly enhance the therapeutic response to immune checkpoint blockade in patients with high‐risk primary and metastatic melanoma, conferring improved overall survival [121, 122]. The positive outcome was associated with increased B cell receptor (BCR) diversity and clonal immunoglobulin heavy and light chain expression, supporting a critical role for B cells in anti‐melanoma immunity within the skin [121, 122]. The GC‐like reaction in tumor TLSs appears to be critical for better patient prognosis because antibody secretion by PCs without B and T cell interaction has been reported as insufficient for tumor clearance [126]. Yet, primary human melanomas containing GC‐like TLSs are rare, but most melanoma metastases contain disorganized TLS distinct from GC‐like structures [127]. Therefore, understanding factors that control the formation of GC‐like TLSs in the human skin is critical for the development of novel therapeutic strategies.

## 5 Therapeutic Potential of Microbiota‐TLO Interactions

Reported evidence that human skin supports B cells, homeostatic antibody secretion, and, under specific conditions, also TLS formation and GC reaction prompts us to entertain the idea of skin as the largest and most accessible platform for non‐invasive delivery of therapeutics to patients. Our findings in murine models demonstrate that immune responses to commensal colonization involve a coordinated T and B cell response that is much stronger than previously anticipated [11, 48, 49]. Mechanistic understanding of the unique strategy developed by the skin to regulate its relationship with the microbiota may, therefore, open the door to a novel type of topical vaccination and immunotherapy approaches.

The therapeutic potential may reside within the microbiome itself [128, 129, 130]. Isolated from healthy human subjects, a unique strain of skin commensal *S. epidermidis* producing 6‐N‐hydroxyaminopurine was shown to display antimicrobial and antitumor activities in mice. Colonization of murine skin effectively reduced the incidence of ultraviolet radiation‐induced tumors, suggesting that the microbiome of some individuals may confer protection against skin cancer [129]. Developing this concept further, experimental models elegantly show that engineering symbionts to prime immunity against designated targets could harness host‐microbiota interactions therapeutically [14, 131]. For instance, skin colonization with a customized mouse melanoma antigen‐expressing *S. epidermidis* triggered an organism‐wide foreign antigen‐specific T‐cell reaction that could eliminate subcutaneous melanoma grafts and lung metastases [131]. Further, similarly engineered symbionts expressing diphtheria or pertussis toxin fragments were shown to elicit a potent, durable, and antigen‐specific antibody response in mice that could protect against a lethal challenge with the respective toxin [14]. It is important to note that skin topical association, even with engineered commensals, is not an invasive nor inflammatory procedure that nevertheless effectively activates lymph node responses and, as we know now, also initiates dermal TLO formation, ensuring profound immunity at systemic and local sites [11, 14]. It remains to be investigated whether the skin's response to engineered *S. epidermidis* is as strong in people as it is in mice, particularly because wild‐type *S. epidermidis* is a permanent and ubiquitous colonizer of human skin [132], whereas in mice it is not part of the natural skin microbiota [133]. As such, it is unclear how preexisting wild‐type‐specific antibody responses in humans will impact colonization and immune activation of engineered commensal [14, 134, 135, 136]. Further, effective skin colonization might prove to be challenging also because engineered bacteria must compete with the host's preexisting core microbiota, which requires exceptionally high bacterial fitness. Vaccine or immunotherapy formulation and logistics chain of distribution must be carefully considered, as it is very difficult to deliver any live, biologically active bacteria in a manufactured product to the patient without compromising on biotherapeutic efficacy. Finally, what will be the long‐term consequences of live and self‐maintaining commensal‐based vaccine or immunotherapy, given that once established, a cutaneous symbiont retains its niche for at least 2 years and, possibly, as long as the lifetime [137, 138]? Would immunity become exhausted, tolerizing, or overactivated and disproportionally biased toward one specific antigen? These questions must be addressed before an engineered commensal‐based therapeutics approach can be used in humans.

One of the major challenges in vaccination is to achieve a high level of antibody responses within barrier tissues with limited inflammation. Microbiota‐induced non‐inflammatory dermal TLOs could present an attractive solution to this obstacle. Using TLO as an immune infrastructure that can be repurposed to generate more specific antibody responses to unrelated therapeutic modalities could open the door to novel barrier vaccination strategies. Dermal TLOs can be induced by any novel to the host skin commensal in adulthood [11]. While it remains to be investigated if human skin contains baseline homeostatic TLOs and can induce *de novo* structures after exposure to a novel colonist, it is important to understand the principles of TLO formation and to decode the highly coordinated interaction between skin immune cells, hair follicles, and microbiota in experimental murine models. The skin tissue might employ specific strategies based on its unique configuration in humans; however, basic components of the cutaneous barrier are shared with mice, allowing for more in‐depth mechanistic understanding. Restructuring resulting microbiota‐specific TLO immune response to highly coordinated GC reaction specific to other types of antigens and vaccine modalities could pave the way for the development of needle‐free vaccines that would be affordable to everyone, easy to distribute, and to administer without involvement by healthcare professionals. Currently used topical therapeutic molecules absorbed by the skin are designed with properties to mimic the skin surface by being lipophilic and slightly acidic, and with a molecular weight less than 500 Da to be able to cross the epidermis [139, 140]. These limitations preclude absorption of most drugs at therapeutic levels. To facilitate transdermal delivery of antigens, microneedle arrays loaded with biotherapeutics, vaccines, and other materials have been used to cross the stratum corneum for direct release into the dermis [141, 142, 143]. However, intradermal discharge of molecules leads to immediate systemic absorption into circulation with the potential to cause off‐target effects.

The central role of epidermal LCs in TLO formation provides an opportunity to retain antigens within skin tissue by avoiding systemic delivery routes. LCs are very reluctant migrants to draining lymph nodes when compared to conventional DCs [144]. More specifically, epidermal LC migration to the dermis is orchestrated by C‐X‐C chemokine receptor type 4 (CXCR4), while the further journey to the lymph node requires CCR7 signaling [145]. Given that dermal TLOs are formed and maintained even in CCR7‐deficient mice [11], temporal local blockade of CCR7 within the dermis could facilitate immune antigen retention at the barrier tissue itself [17, 146, 147]. Given that the TLO‐associated dermal GC reaction provides both local and systemic humoral output, such an approach will not compromise host protection but instead prevent any possible adverse events. TLO association with the hair follicle provides a unique gateway for specific antigen delivery and LC targeting [148, 149]. Hair follicles serve as a reservoir and a pathway for LC trafficking in between the epidermis, particularly in response to external irritation [28]. The immunological environment at the center of homeostatic dermal TLO allows for transfollicular delivery of therapeutics packaged in nanoparticles for an immediate humoral response in a non‐invasive manner [26, 150], coining the hair follicle as nature's needle in a needle‐free immunization. Recently, targeted drug delivery via hair follicles has become a very appealing research strategy for treating hair follicle‐related disorders, including androgen‐associated diseases, hair loss, and acne vulgaris [151, 152, 153]. However, clinical implementation is rare, and the current experimental challenges focus on effective drug formulations with particular interest in delivery and pharmacodynamics [154]. For the needle‐free immunization strategy, the greatest challenge might be to precisely target a TLO‐associated follicle. Although topical microbial colonization effectively stimulates the formation of these structures, not every follicle is associated with TLO in any given colonized area [11]. We speculate that a critical biomass of stimulating antigen must be achieved within the follicle to unleash the immune priming in this tightly controlled microenvironment. Indeed, in mice, TLOs were repeatedly observed in areas at the very base of the pinna, where tissue curvature is more pronounced, allowing for prolonged exposure and better accumulation of symbionts, resulting in more effective microbial colonization [11]. In addition, a tightly controlled microenvironment with structural and immunoregulatory components as well as the maturation status of the follicle might impact TLO formation. Further research is needed to dissect these tissue intrinsic circuits. A possible strategy for needle‐free topical vaccination could be a highly controlled and optically monitored two‐step process where *de novo* TLOs are induced in a defined and restricted skin area prior to the follicular immunization, aiding a predictable and successful outcome [154]. Importantly, physiological features of the hair follicle, such as the natural hair cycle, shedding, or sebum secretion, could also allow for the local release of therapeutic TLO‐derived antibodies to the surface of the skin, combating pathogen attack and ensuring the overall first line of immune defense. Therefore, exploring the mechanism of commensal‐derived antigen delivery and/or capture within the skin may facilitate innovative therapeutic development and treatment of topical diseases.

While GCs are vital for building robust immune responses, in certain situations, an active GC reaction may be harmful to the host. A rapid mutation rate and a dysregulated selection process can lead to the development of B cells that are either autoreactive or cancerous, giving rise to antibody‐mediated skin diseases or cutaneous lymphomas [155, 156, 157]. Hair follicles could serve as a delivery route to the inhibitors of the GC reaction when inflammatory TLS and homeostatic TLO activity in the dermis are no longer needed. In addition to known TLS‐associated skin pathologies such as HS and pemphigus vulgaris, dampening of the skin's humoral immunity might also be required for homeostatic antibody production to specific symbionts at a given time. As an example, gene editing therapies for rare diseases may use Cas9 orthologs derived from skin‐homing microbes *Staphylococcus aureus* and *Streptococcus pyogenes* [158]. A healthy human population has very high serum reactivity to these Cas9 variants [159, 160]. This can be explained by the fact that *S. aureus* and *S. pyogenes* are frequent human skin commensals with opportunistic pathogen tendencies that colonize approximately 20% and 40% of the population, respectively, at any given time [161, 162]. Significant pre‐existing antibody titers to Cas9 gene therapies might have derived from both classical GC response in lymph nodes or dermal TLOs due to systemic infection and homeostatic exposure with respective microbe. Gene therapy administered to a patient with preexisting immunity might cause life‐threatening complications; therefore, exploring potential ways to fine‐tune microbiota‐specific TLO output at a certain time could be one way to improve existing treatments. It has been shown that the inhibition of the interleukin 27 (IL‐27) pathway dampens T_H_17‐rich TLSs in early arthritis [163]. Dermal TLOs are sensitive to the loss of critical GC factors such as the IL‐21 signaling pathway, cognate T cell‐ B cell interactions via CD40L, and T_FH_ cells, providing potential targets for time‐controlled immune inhibition and structural disruption [11]. Yet, every individual TLO and TLS composition is slightly different based on the stimulating microbe, disease context, and tissue microenvironment. Therefore, identifying underlying mechanisms in TLO induction might help develop a universal strategy where dermal GC activity can be clinically manipulated to meet the patient's needs for better therapeutic outcomes.

## 6 Outlook

The skin is one of the largest organs serving as a critical barrier against the harsh extrinsic environment. Its core function comes in various forms of host protection that range from preventing desiccation, chemical damage, and hypothermia to mounting a highly specific immune attack against invading pathogens. Lifelong cohabitation with commensal microorganisms controls and educates the host's immune system, yet any perturbations to normal skin homeostasis can cause microbial dysbiosis and increase infection risk. The skin has adapted to mitigate these risks by establishing dermal TLOs as isolated autonomous humoral factories that control the homeostatic equilibrium of cutaneous microbiota and tissue barrier integrity through protective antibody production. The very essence of TLO formation, maintenance, and function resides within the skin's unique morphology that allows for commensal colonization of appendages such as hair follicles that are surrounded by LCs. Decoding of the highly coordinated interaction between LCs, hair follicles, and the microbiota could not only broaden our understanding of host‐microbiota interactions but also open the door to novel vaccination modalities based on the mimicry of these physiological “needles”. To evaluate potential strategies of skin‐based topical therapies, we should more thoroughly investigate determinants that ensure the survival of cutaneous commensals within a follicular niche, antigen capture and processing by the LCs, and priming of skin intrinsic adaptive immune response. Furthermore, mechanisms of antibody transport topically to the skin surface and systemically, as well as factors that dictate TLO maintenance, regulation, and survival, will need to be elucidated in more detail.

A critical role of B cells in skin health is no longer only ascribed to inflammatory conditions, but also clearly sets the stage for homeostatic surveillance of the physical cutaneous border. The magnitude of its impact on human skin protection and specific phenotype and features of B cells associated with the skin in various microbial settings, and their ability to form dermal TLOs at different stages of the host's life, remain to be clarified. These lymphoid niches should be considered therapeutic hotspots that could enable specific B cell‐mediated topical treatments or immunization approaches. On the downside, if dysregulated and uncontrolled, B cell mutagenesis could enable continuous inflammation, facilitating homeostatic TLO conversion into inflammatory TLS and subsequent skin autoimmunity or malignancy. Due to growing evidence of B cell roles in cutaneous pathologies, therapeutic strategies based on personalized, precise characterization of cutaneous leukocytes must be advanced to target dermal TLSs for clinical applications.

At the dawn of a new era in experimental medicine, research funders and regulatory agencies around the globe demand replacing animal testing in the development of clinical therapies with more effective, human‐relevant methods. Although recapitulating a fully living organism in a culture system is impossible, the development of ex vivo human skin models is ongoing [164]. Such an approach allows for direct analysis of tissue response to topical therapy [165], cytotoxic and inflammatory stimuli [166], and, using a 3D healthy skin model, assessment of B cell infiltration from systemic sclerosis patients [167]. The major challenge in using ex vivo models is to fully replicate the complex cellular and humoral interactions and processes of the human immune system, including that of GC reaction [168]. For this reason, and until better models are available, many tools and experimental approaches, including limited laboratory animal testing, patient‐derived skin organoids, in vitro cell culture systems, and advanced computer simulations, should be used to answer the outlined questions.

Collectively, our work uncovers a previously unappreciated concept of skin's immune autonomy through microbiota‐tailored humoral immunity that effectively safeguards the cutaneous border and launches the first line of immune defense [11]. At this point, the questions outnumber the answers, and more extensive research is needed to fully understand the uncharted field of skin's humoral immunity. For those who are brave enough to embark on the journey, this line of research will bring broad implications for our understanding of skin physiology, immunity, and inflammatory disorders.

## Conflicts of Interest

The author declares no conflicts of interest.

## Data Availability

The author has nothing to report.
